# Involvement of BIG5 and BIG3 in BRI1 Trafficking Reveals Diverse Functions of BIG-subfamily ARF-GEFs in Plant Growth and Gravitropism

**DOI:** 10.3390/ijms20092339

**Published:** 2019-05-11

**Authors:** Shan Xue, Junjie Zou, Yangfan Liu, Ming Wang, Chunxia Zhang, Jie Le

**Affiliations:** 1 Key Laboratory of Plant Molecular Physiology, CAS Center for Excellence in Molecular Plant Sciences, Institute of Botany, Chinese Academy of Sciences, Beijing 100093, China; xueshan1984@yeah.net (S.X.); zoujunjie@caas.cn (J.Z.); liuyangfan@ibcas.ac.cn (Y.L.); ming.wang@genefirst.com (M.W.); chunxia.zhang@ibcas.ac.cn (C.Z.); 2 University of Chinese Academy of Sciences, Beijing 100049, China

**Keywords:** ARF protein, BIG family ARF-GEF, auxin, brassinosteroids, gravity response

## Abstract

ADP-ribosylation factor-guanine nucleotide exchange factors (ARF-GEFs) act as key regulators of vesicle trafficking in all eukaryotes. In Arabidopsis, there are eight ARF-GEFs, including three members of the GBF1 subfamily and five members of the BIG subfamily. These ARF-GEFs have different subcellular localizations and regulate different trafficking pathways. Until now, the roles of these BIG-subfamily ARF-GEFs have not been fully revealed. Here, analysis of the *BIGs* expression patterns showed that *BIG3* and *BIG5* have similar expression patterns. *big5-1* displayed a dwarf growth and *big3-1 big5-1* double mutant showed more severe defects, indicating functional redundancy between *BIG3* and *BIG5*. Moreover, both *big5-1* and *big3-1 big5-1* exhibited a reduced sensitivity to Brassinosteroid (BR) treatment. Brefeldin A (BFA)-induced BR receptor Brassinosteroid insensitive 1 (BRI1) aggregation was reduced in *big5-1* mutant, indicating that the action of BIG5 is required for BRI1 recycling. Furthermore, BR-induced dephosphorylation of transcription factor BZR1 was decreased in *big3-1 big5-1* double mutants. The introduction of the gain-of-function of *BZR1* mutant *BZR1-1D* in *big3-1 big5-1* mutants can partially rescue the *big3-1 big5-1* growth defects. Our findings revealed that BIG5 functions redundantly with BIG3 in plant growth and gravitropism, and *BIG5* participates in BR signal transduction pathway through regulating BRI1 trafficking.

## 1. Introduction

ADP-ribosylation factors (ARFs), are small G proteins that function in endomembrane trafficking. In plants, ARF-GEFs can be classified into GBF1 and BIG subfamilies [1,2]. In Arabidopsis, reports have shown that GNOM and GNOM-LIKE1, members of GBF1-subfamily, are involved in internal trafficking of auxin transporter PIN-FORMED (PIN) proteins [3,4,5,6,7,8,9] and brassinosteroid (BR) receptor BRI1 [10,11]. However, the functions of BIG-subfamily ARF-GEFs (BIG1–BIG5) in plant growth and development have not been fully investigated. Previous studies revealed that *BIG5/MIN7/BEN1* plays important roles in pathogen defense [12] and root growth [13,14]. The *ben1* mutants had short roots and displayed defective polar distribution of PIN1 and PIN2, affecting PIN-mediated auxin transport for organ development and gravitropism [4,15]. However, whether and how BIG5 and other members of BIG subfamily take part in plant growth and gravitropic response remain unknown.

Brassinosteroids (BRs), known as important plant hormones, play important roles in a variety of developmental processes, especially in controlling plant organ size, regulating shoot and root gravitropism [16,17,18,19,20,21,22]. BRs play negative role in Arabidopsis hypocotyl gravitropism [23,24]. Exogenous BRs treatment can dramatically reduce root growth [16,25,26] and enhance root tip deviation from vertical direction [27,28]. BRI1 acts as a major receptor of BRs and mutation of *BRI1* leads to extremely dwarf phenotype and reduced sensitivity to BR response [29,30,31]. BRI1 overexpression lines (*BRI1-GFP OE*) have similar phenotype as exogenous BR treatment, such as enlarged rosette leaf size [32,33,34,35]. BRs bind to the extracellular domain of BRI1 [36], and activate subsequent downstream signaling pathway, leading to the dephosphorylation of transcription factors BZR1 and BES1 by PP2A [37,38]. The dephosphorylated BZR1 and BES1 then enter into nucleus and regulate expression levels of BR responsive genes [17,37,39,40,41,42,43]. 

BRI1 is mainly localized at the plasma membrane (PM) and undergoes constitutive endocytosis from PM to the trans-golgi network (TGN) or early endosome (EE), where it is either recycled to the plasma membrane or sorted for degradation in the vacuoles via the MVBs [44,45,46]. BRI1-GFP is almost completely co-localized with endocytic tracer FM4-64, closely co-localized with golgi marker ST-GFP [47,48], and partially co-localized with TGN marker VHA-a1-GFP [10,11]. Previous research found that Brefeldin A (BFA), a vesicle transport inhibitor produced from fungal organisms, can disrupt BRI1 cycling between PM and TGN or EE, reducing the dephosphorylation levels of BES1 [10,11]. Whether members of BIG subfamily participate in BR signaling transduction remains unclear.

In this study, we analyzed the expression patterns of these *BIG* genes and their roles in plant growth and development. Our results showed that *BIG3* and *BIG5* function redundantly in controlling plant size and regulating BR signaling. The *big3-1 big5-1* double mutants displayed more severe growth defect than *big5-1* single mutant. The *big5-1* mutant exhibited accelerated gravity responses and reduced sensitivity to BR. The trafficking of BR receptor BRI1 was restrained in *big5-1* mutant. Furthermore, the dephosphorylation level of BZR1 was decreased in BR-treated *big5-1* compared to wild-type plants. These results showed that BIG5 functions in regulating plant growth and gravitropism partially through mediating BRI1 recycling and subsequent BR signaling transduction pathway.

## 2. Results

### 2.1. BIG5 and BIG3 Share a Redundancy Function in Controlling Rosette Leaves and Inflorescence Development in Arabidopsis

**Figure 1:**
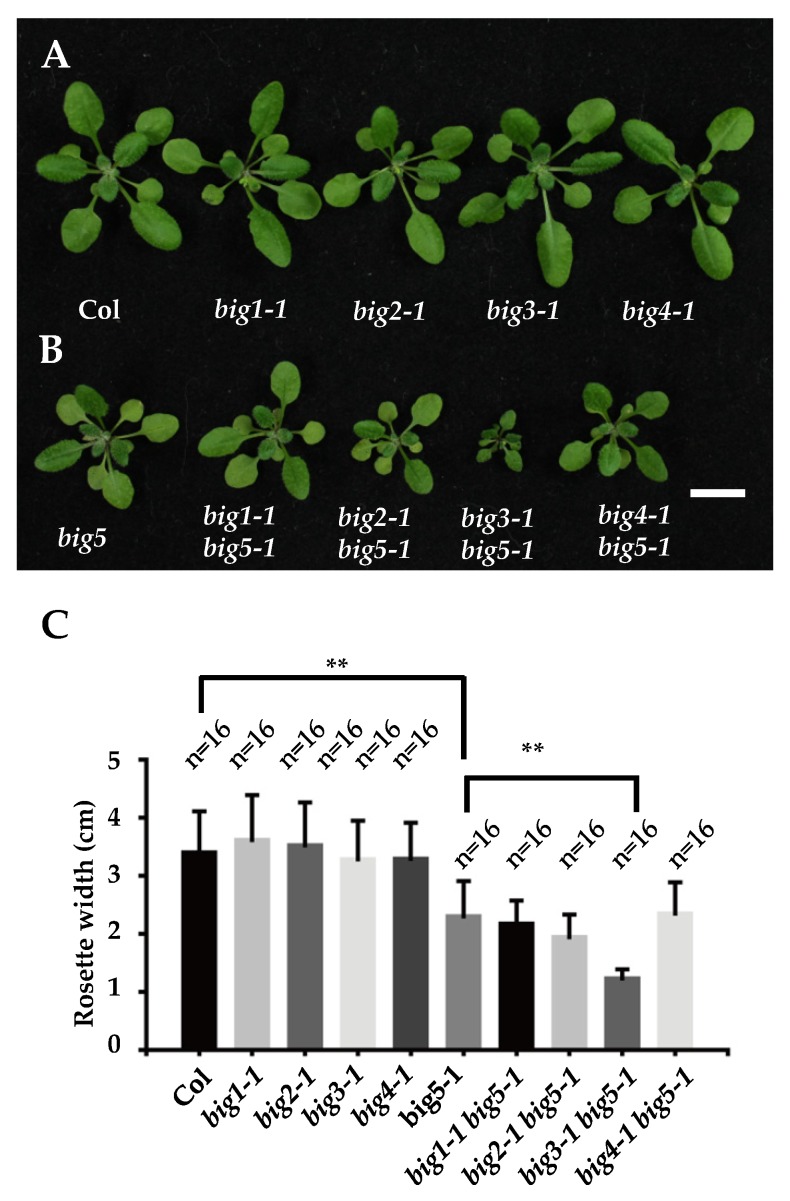
Growth phenotypes of BIG-subfamily mutants. (**A**) Overall growth 4-week-old seedling of BIG-subfamily single mutants, *big1-1*, *big1-2*, *big3-1*, *big4-1* exhibit similar size with Col. (**B**) *big5-1* shows a reduced rosette size. *big1-1 big5-1*, *big2-1 big5-1*, and *big4-1 big5-1* double mutants have a similar seedling size with *big5-1*. Only *big3-1 big5-1* double mutant shows an aggregated growth defects. (**C**) Quantitative analysis of rosette width. Bars = 1 cm. Error bars represent standard deviations, significant difference after Student’s *t*-test, ** *p* < 0.01.

The BIG ARF-GEF subfamily is conserved among mammals, yeast, and plants. Both mammals and yeasts have two BIG genes, whereas the Arabidopsis genome encodes five BIG family members. To analyze their roles in plant growth and development, mutants of these genes were acquired, identified, and used for phenotype screening (Figure S1). Under normal growth conditions, the *big1-1*, *big2-1*, *big3-1*, and *big4-1* mutants showed similar overall seedling size compared to wild-type plants (Figure 1), whereas, the *big5-1* mutant had smaller overall growth size compared to wild-type plants, displaying reduced rosette leaf size and inflorescence height (Figure 1 and Figure S2A–K). 

Phylogenetic analysis of BIG subfamily showed that these five members can be divided into three groups: BIG1/4 group, BIG2/3 group, and BIG5 (Figure S2L). To test whether functional redundancy exists between BIG5 and other members, we then crossed *big5-1* mutant with *big1-1*, *big2-1*, *big3-1*, *big4-1, and* obtained corresponding double mutants, respectively. When comparing *big5-1* single mutant with other double mutants in *big5-1* background, the *big3-1 big5-1* showed more severe growth defect than *big5-1*, displaying much smaller size of rosette leaves and shorter inflorescence height than *big5-1*. These results indicated that BIG3 and BIG5 function redundantly in determining plant organ size (Figure 1B,C and Figure S2F–K).

### 2.2. Complementation of big5-1 Growth Defect with BIG5-GFP and BIG5^M731L^-GFP 

**Figure 2:**
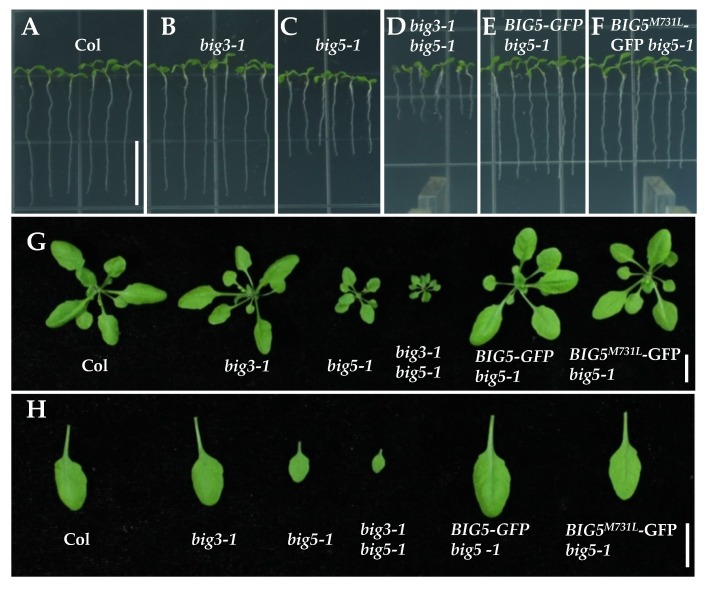
Both *BIG5-GFP* and *BIG5^M731L^-GFP* complement *big5-1* defective overall plant growth. (**A**–**D**) Seedlings *big5-1* and *big3-1 big5-1* mutants show a reduced primary root length. By contrast, *big3-1* has a normal root. (**E**,**F**) Both wild-type *BIG5-GFP* and *BIG5^M731L^-GFP* transgenic lines could rescue *big5-1* growth defects. (**G**–**H**) The sizes of rosette leaves of 4-week-old *big5-1* and *big3-1 big5-1* mutants are much smaller than Col and *big3-1*. While, *BIG5-GFP* and *BIG5^M731L^-GFP* fully rescued *big5-1* growth defects. Bars = 1.5 cm in (**A**–**F**), 1 cm in (**G**) and (**H**).

To confirm the function of *BIG5* in regulating plant growth, we constructed a wild-type *BIG5-GFP* and a BFA-resistant version mutant *BIG5^M731L^-GFP*, and then introduced these constructs into wild-type plants by floral dip method [49]. Then the transgenic lines were crossed with *big5-1* mutant, respectively. When introducing *BIG5-GFP* or *BIG5^M731L^-GFP* into *big5-1* mutant, the transgenic lines harboring comparable expression level of *BIG5-GFP* and *BIG5^M731L^-GFP* were selected (Figure S3H). The growth defects of *big5-1* were completely rescued in *BIG5-GFP big5-1* and *BIG5^M731L^-GFP big5-1* plants, displaying normal primary root length (Figure 2A–F) and similar rosette leaf size (Figure 2G–H) as that of wild-type plants, indicating that BIG5, together with BIG3, plays an important role in plant growth, including root growth, rosette leaf size, and inflorescence height. 

### 2.3. The Expression Patterns and Subcelluar Localization of BIG5 in Arabidopsis

To determine BIG5 subcellular localization, fluorescence signals in the root epidermal cells of *BIG5-GFP* and *BIG5^M731L^-GFP* transgenic plants were checked. BIG5-GFP (Figure S4A–C), and BIG5^M731L^-GFP (Figure S4D–F) green fluorescences have partial co-localizations with TGN marker VHaA1-mCherry red fluorescences, indicating that BIG5 has partial TGN localization and may have a conserved function in regulating endomembrane trafficking.

**Figure 3:**
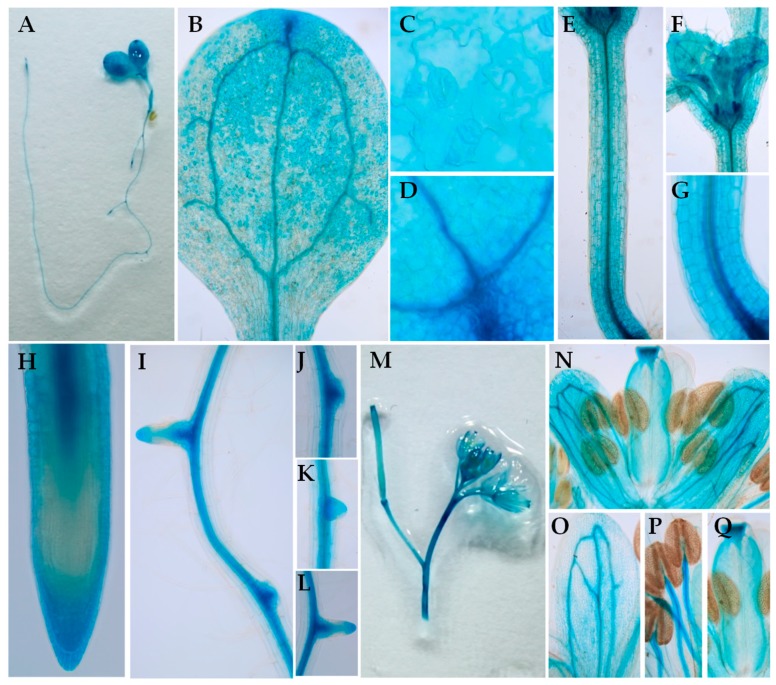
The expression pattern of *BIG5* in Arabidopsis. (**A**–**Q**) The expression patterns of *pBIG5:*GUS in 7 dag Arabidopsis seedling (**A**), cotyledon (**B**), epidermal cells (**C**), vascular structure (**D**), a hypocotyl (**E**), young leaves (**F**), stele cells (**G**), root tip (**H**), lateral roots (**I**–**L**), and flowers (**M**–**Q**).

To determine the expression patterns of BIG subfamily genes, transgenic plants harboring a β-glucuronidase (GUS) reporter gene driven by their native promoters were constructed and transformed into wild-type plants, respectively. GUS staining assays showed that these five *BIG* genes display different expression patterns (Figure S5). As for *BIG5*, GUS staining of the *BIG5:GUS* transgenic plants revealed that *BIG5* is universally expressed in whole plants including roots, cotyledon, rosette leaves, hypocotyl, and flowers (Figure 3 and Figure S5U–Y). Furthermore, *BIG3* (Figure S5K–O) had a similar expression pattern as *BIG5* in root tips, hypocotyl, and leaves. The expression level of BIG family gene in roots has been analyzed by RT-qPCR, and the results showed that *BIG3* and *BIG5* have relative high expression levels compared to other BIG family genes (Figure S3I). The similar expression patterns between *BIG3* and *BIG5* indicated their functional redundancy in regulating plant growth (Figure 1 and Figure S2). 

### 2.4. The big5-1 Mutant Displayed Abnormal Gravitropic Response and Was Insensitive to BR Treatment

**Figure 4:**
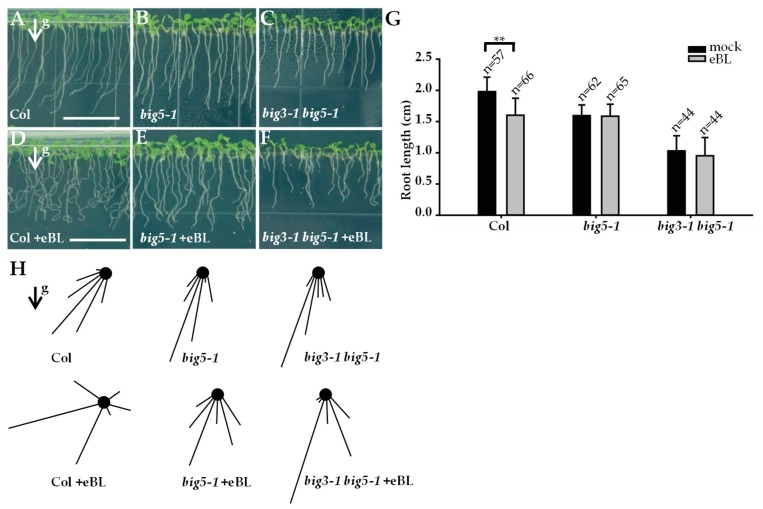
BIG5 and BIG3 are required for root elongation and gravitropic response. (**A**–**C**) Vertically grown seedlings of Col (**A**), *big5-1* (**B**), and *big3-1 big5-1* (**C**). (**D**–**F**) Seedings grown on medium supplemented with 10 nM eBL. In contrast to the twisted and significantly reduced total root length in Col (**D**), *big5-1* (**E**) and *big3-1 big5-1* (**F**) are insensitive to BR treatment. (**G**) Quantitative analysis of total root length. (**H**) Arrows indicate the gravity vector. Vector-bar graphs represent the degree of root tip deviation from the gravity vector. Bars = 1.5 cm; Error bars represent standard deviations, significant difference after Student’s *t*-test, ** *p* < 0.01.

The root length of *big5-1* mutant was much shorter than that of wild-type plants (Figure 4A,B), consistent with previous research on *ben1* mutant [13]. Furthermore, the root length of *big3-1 big5-1* double mutants was much shorter than that of *big5-1* single mutant (Figure 4B,C). Previous reports revealed the important role of GBF1 ARF-GEF in gravitropism [3]. Under vertical growth condition, the root tips of *big5-1* and *big3-1 big5-1* showed reduced deviation angle from gravity vector compared to Col (Figure 4A–C,H). After a 90° reorientation of the roots, root curvature was measured at indicated time points. Interestingly, the *big5-1* mutant displayed accelerated root curvature compared with wild-type plants, and *big3-1 big5-1* double mutants showed enhanced root gravitropic response than *big5-1* single mutant (Figure S6). These results indicated that *BIG5*, together with *BIG3*, plays negative roles in root gravitropic response. 

To determine whether *BIG5* participates in BRs-mediated root gravitropic response, *big5-1*, *big3-1 big5-1*, and wild-type plants grew vertically on half-strength MS medium supplemented with 10 nM eBL, and root angle deviation from vertical was then analyzed. Treatment with eBL resulted in reduced root length of Col, whereas eBL had no effect on root growth of *big5-1* and *big3-1 big5-1* mutants (Figure 4G). Moreover, eBL treatment induced an enhanced root deviation from gravity vector in Col, showing twisted root growth (Figure 4D). By contrast, *big5-1* and *big3-1 big5-1* were insensitive to eBL treatment, showing dramatically reduced twisted roots (Figure 4E,F,H). 

We further investigated the role of BIG5 in BR-mediated shoot gravitropism. When grown in the dark, application of eBL inhibited the elongation of hypocotyl and induced twisted hypocotyl growth in Col. By contrast, *big5-1* hypocotyls showed reduced sensitivity to eBL treatment (Figure S7A–E). Previous reports showed that sucrose could alleviate BR effects on hypocotyl gravitropic growth [23,24]. In the absence of sucrose, BR can disrupt the uniform direction of hypocotyl growth of Col in dark. Exogenous sucrose restored hypocotyl negative gravitropism in the BR-treated Col seedlings. However, exogenous sucrose had no additional effect on hypocotyl growth orientation of *big5-1* in the presence of eBL (Figure S7F–N). Furthermore, we tested the effect of eBL on hypocotyl length under light conditions. The results showed that the hypocotyl length of Col was obviously elongation induced by eBL, while the *big3-1 big5-1* show reduced sensitivity to eBL (Figure S3A–E). These results indicated that BIG5 is required for BR-mediated gravitropic responses. 

### 2.5. BIG5 and BIG3 Are Required for BRI1 Trafficking 

Arabidopsis GNOM and GNOM-LIKE1 are involved in PIN proteins trafficking during auxin signal transduction [7,8]. BIG5 and BIG3 may participate in BR signal transduction through regulating BR receptor trafficking. FM4-64 is a membrane-selective dye and widely used to study endocytosis, vesicle trafficking and organelle organization in living eukaryotic cells [50]. We then used FM4-64 to monitor endocytosis in root epidermal cells of Col, *big5-1*, and *big3-1 big5-1* double mutants. As shown in Figure S8, after staining with FM4-64 for 30 min, there were substantial numbers of punctuated fluorescent vesicles in the cytosol of Col root epidermal cells, whereas, there were only a few fluorescent vesicles detected in the root epidermal cells of *big5-1* or *big3-1 big5-1* double mutants, indicating that endocytosis was defective in the *big5-1* and *big3-1 big5-1* double mutants.

**Figure 5:**
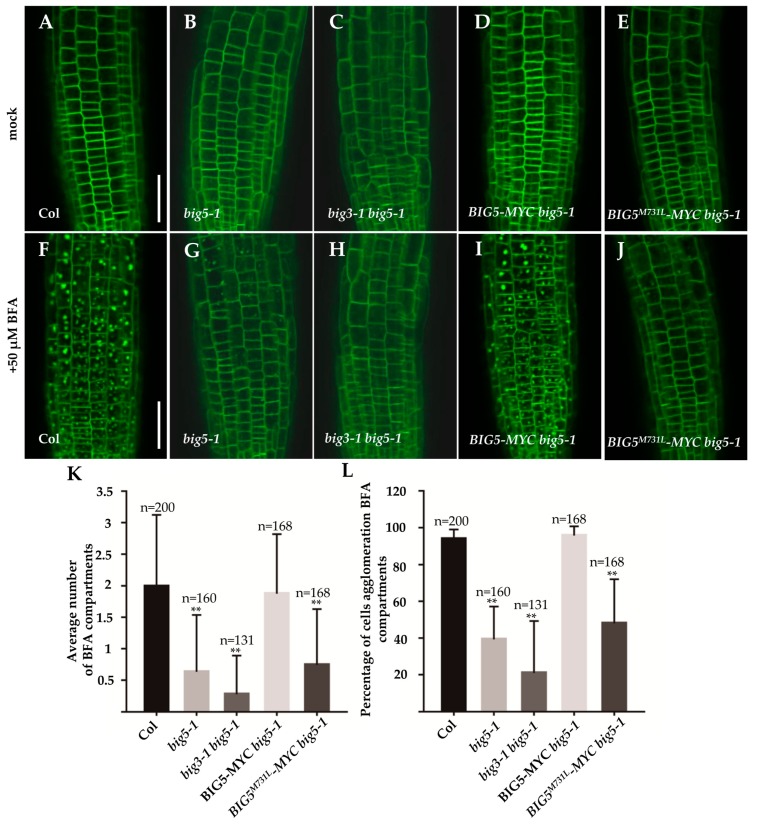
Aggregation of BRI1-GFP in BFA-compartments is blocked in *big5* and *big3 big5* mutants. (**A**–**E**) BRI1-GFP predominantly localize on plasma membrane. (**F**) BFA induced aggregation of BRI1-GFP in BFA-compartments. (**G**,**H**) No trapped BRI1-GFP signals found in both *big5-1* (**G**) and *big3-1 big5-1* (**H**) after BFA treatment. (**I**) BIG5-MYC restores the BRI1-GFP aggregation in BFA-compartments in *big5-1*. (**J**) By contrast, BFA-resistant version *BIG5^M731L^-MYC* complemented *big5-1* growth defects but failed to induce BRI1-GFP aggregation after BFA treatment. (**K**) Number of BFA-compartments per epidermal cell. (**L**) Quantitative analysis of the percentage of cells showing BFA-compartments. Bars = 40 µm. Error bars represent standard deviations, significant difference after Student’s *t*-test, ** *p* < 0.01.

The fungal toxin BFA acts as a vesicle transport inhibitor that inhibits protein trafficking in the endomembrane system, leading to the formation of BFA compartments [50]. We then used BFA to monitor BRI1-GFP localization in wild-type, *big5-1*, and *big3-1 big5-1* backgrounds (Figure 5). After treatment with 50 μM BFA for 30 min, BRI1-GFP aggregated into BFA-compartments in Col and *big5-1* mutant. Interestingly, Col accumulated more and larger BRI-GFP BFA compartments than *big5-1* mutant (Figure 5G) in the root epidermal cells. However, BFA compartments were hardly detected in *big3-1 big5-1* mutants (Figure 5H). When *BIG5-MYC* construct was introduced into *big5-1* mutant, there was no obvious difference in accumulated BFA compartments between Col and *BIG5-MYC big5-1* (Figure 5I,K,L). These results suggested that BIG5 participates in BR receptor BRI endocytosis.

Amino acids in the SEC7 domain are rather conserved between ARF-GEFs, and previous research proved that Met within the conserved SEC7 domain is one of the key residues for BFA sensitivity [5,7,8]. In BIG5, the Met is located at 731 within SEC7 domain (Figure S4H). In Arabidopsis, most ARF-GEF proteins are predicted to be sensitive to BFA treatment, except for BIG3 and GNOM-LIKE1 [7,8,9]. So it is hardly to distinguish whether other ARF-GEF proteins are also involved in BRI1 recycling. To better understand this complex process, we constructed a BFA-resistant version of *BIG5*, *BIG5^M731L^-MYC*, in which the Met^731^ within the conserved SEC7 domain was substituted with Leu^731^. As predicted, BFA treatment induced fewer and smaller BFA compartments in BIG5^M731L^ *big5-1* than *BIG5-MYC big5-1* (Figure 5J–L). These results indicated that BIG5 is indeed a BFA sensitive ARF-GEF and Met^731^ is essential for BIG5′s BFA sensitivity. As BIG3 is a BFA-insensitive ARF-GEF, BIG5 might play a predominant role in mediating BRI1 endocytosis and recycling. Taken together, pharmacologic and genetic results suggested that both BIG5 and BIG3 are involved in BR receptor BRI1 trafficking. 

### 2.6. The Loss-of-Function of BIG5 Results in a Reduced BR Sensitivity 

**Figure 6:**
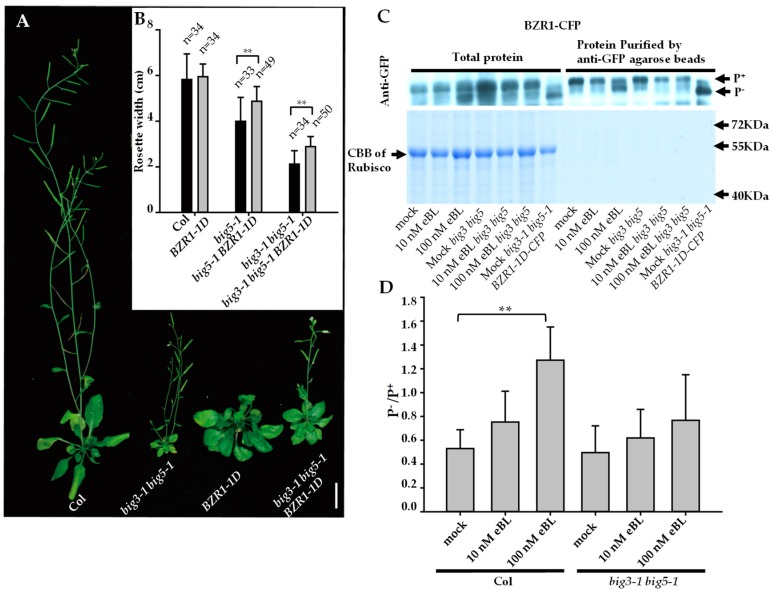
BIG5 and BIG3 act upstream of transcription factor BZR1 in BR signaling pathway. (**A**–**B**) Gain-of function *BZR1-1D* partially rescued the *big3-1 big5-1* phenotype in plant size (**A**). Quantitative analysis of rosette width (**B**). (**C**) Western blot analysis of the BZR1-CFP dephosphorylation in 7 dag *big3-1 big5-1* double mutant and WT (Col-0) in the presence or absence of eBL. In Col the BZR1 dephosphorylation level obviously increased as gradient eBL added, while the dephosphorylation BZR1 in *big3-1 big5-1* failed to response to eBL. By contrast, gain-of-fucntion *BZR1-1D-CFP* conferred the dephosphorylation in *big3-1 big5-1* mutants. The bottom box is CBB gel showing the Rubisco proteins as the loading control. (**D**) Quantitative analysis of the percentage of dephosphorylated BZR1 relative to the total BZR1 detected. Bar = 2 cm**.** Error bars represent standard deviations, significant difference after Student’s *t*-test, ** *p* < 0.01.

As BIG5 functions in regulating BR receptor BRI1 trafficking, mutation of *BIG5* may impair BR downstream signal transduction. The *big5-1* mutant showed obviously reduced growth including rosette leaf size and inflorescence height, which is similar as the dysfunctional mutant of *BRI1*. To assay the genetic relationship between *BRI1* and *BIG5*, we crossed *big5-1* and *big3-1 big5-1* with *BRI1* overexpression line (*pBRI1:BRI1-GFP, BRI ox*) and the BR downstream transcription factor *BZR1* gain-of-function mutant *BZR1-1D*, respectively. Overexpression of *BRI1* induced enlarged rosette leaves, whereas this effect was suppressed in *big5-1* or *big3-1 big5-1* background (Figure S9A). In *big3-1 big5-1 BZR1-1D* triple mutants, the gain-of-function of *BZR1-1D* partially rescued *big3-1 big5-1* growth defect in rosette leaves (Figure 6A,B). We noticed that the height of the inflorescence in *big3- big5-1* is not rescued, a reasonable explanation for this phenotype is that the *BIG3* and *BIG5* genes are not only involved in BR signal transduction but also in other regulation pathways, such as in auxin signal transduction pathway. These results suggested that BIG5 and BIG3 are required for regulating BRI1 activity and BR-mediated downstream signal transduction. 

In the presence of BRs, BRs can direct interact with BRI1 and activate the BRI1 signaling pathway. BR-induced dephosphorylated BZR1 and BES1 by PP2A can dissociate from 14–3–3 proteins and accumulate in the nucleus to regulate the expression levels of downstream genes [38]. We measured the expression levels of BR responsive gene markers *CPD* and *DWF4* [46,51] in Col and *big3-1 big5-1* with or without 100 nM eBL treatment. The results showed that the expression levels of *CPD* and *DWF4* were obviously decreased in Col after eBL treatment, whereas *CPD* and *DWF4* showed a reduced response to eBL in *big3-1 big5-1* mutant (Figure S3F). To further verify the role of BIG5 in BR signaling network, the dephosphorylation levels of BZR1 in Col and *big3-1 big5-1* double mutants without or with eBL treatment were analyzed. The *BZR1-CFP* construct was introduced into Col and *big3-1 big5-1* double mutant, respectively. Furthermore, Anti-CFP was used to detect phosphorylated or dephosphorylated BZR1-CFP. In Col background, the dephosphorylation level of BZR1 was enhanced with the increase of eBL, showing higher ratio of dephosphorylated BZR1 to phosphorylated BZR1 (P^−^/P^+^) in the presence of 100 nM eBL (Figure 6C,D). In *big3-1 big5-1* background, however, the level of dephosphorylated BZR1 was much lower than in Col in the presence of indicated eBL, showing lower P^−^/P^+^ ratio. These results indicated that disruptions of *BIG3* and *BIG5* affect BR induced dephosphorylation of BZR1 (Figure 6C,D). We also used Bikinin (a non-steroidal, ATP-competitive inhibitor of plant GSK-3/Shaggy-like kinases and can activate BR signaling) [37,52] to analyze BZR1-CFP dephosphorylation levels in Col and *big3-1 big5-1* double mutant. We found that Bikinin can successfully induce dephosphorylation levels of BZR1 in Col and *big3-1 big5-1* double mutants (Figure S3G). These results proved that the BR downstream factor BZR1 in *big3-1 big5-1* still can be active by GSK-3/Shaggy-like kinases. Altogether, these results suggested that the loss-of-function of *BIG5* attenuates BR response mainly through affecting BRI1 trafficking and reducing dephosphorylation level of BZR1, leading to decreased BR response. 

## 3. Discussion

Previous studies have demonstrated that the BR signaling greatly depends on the subcellular recycling of BRI1. The trapped BRI1 in BFA-compartments or genetic mutation of ARF-GEFs (i.e., *gnom*, *gnl1*) promote the dephosphorylation level of BZR1 and enhance the BR signaling as the consequence [10]. In this study, we found that *big5-1* single mutant displayed retarded growth and reduced sensitivity to BR treatment. Furthermore, no obvious phenotype was found in other BIG-subfamily gene single mutants. The *big3-1 big5-1* double mutants showed more severe growth defects and enhanced gravitropic responses than *big5-1* single mutant, revealing that *BIG3* and *BIG5* function redundantly in terms of plant organ size and gravitropism. 

Recently, BIG-subfamily ARF-GEFs, BIG1–BIG4 had been proved to function in regulating post-golgi trafficking, which play an important role in establishing apical and basal polarities [9,53], and act redundantly in ethylene-mediated hook development [54]. Here, we analyzed the subcellular locations of BIG5 and *BIG5^M731L^*, and their sensitivities to BFA treatment. Our results showed that BIG5 partially co-localizes with TGN marker VHA-a1-GFP, consistent with previous observation [13,14]. In addition, the *BIG5^M731L^* showed similar complementary effect as wild-type *BIG5* on rescuing *big5-1* mutant growth defects. However, BRI1-GFP in *BIG5^M731L^* display a BFA-resistant phenotype, indicating the specific role of BIG5 in regulating BRI1 trafficking. 

In previous reports, PIN1 localization is disrupted in *ben1-1* and *ben1-2* mutants, whereas these two alleles have no defects in root gravitropism [13]. The *ben1-2* is a T-DNA-tagged homozygous mutant where the T-DNA is located in the C-terminal of *BIG5* [13]. Different from previous study, our results revealed that the *big5-1* mutant showed accelerated gravitropic response. In *big5-1* mutant, the T-DNA is located in the first intron of *BIG5* genomic sequence (Figure S1A). We compared the *BIG5* transcription levels in *ben1-2* and *big5-1* (Figure S1H,I). The difference in transcription levels between *big5-1* and *ben1-2* may explain their different responses to gravitropic stimulus. In *ben1-1* mutant, a nucleotide substitution from C to T is predicted to create a premature stop codon, disrupting the SEC7 domain. Accordingly, a further work will be needed to characterize the different responses to gravitropic stimulation between *big5-1* and *ben1-1*. Considering the possible redundancy of other BFA sensitive ARF-GEFs, i.e., GNOM, which shows a dominant function in gravity response, it would be interesting to test the gravity response in BFA insensitive forms of both *BIG5^M731L^* and *GNOM^M696L^*. Although our results demonstrated that BIG5 is involved in the aggregation of BRI1-GFP in BFA-compartments using a high concentration of 50 µM BFA, the role of BIG5 in vesicle trafficking needs further investigation.

The introducing of BRI1 OX or *BZR1-1D* could not fully complement *big5-1* growth defects, indicating that BIG5 might be not only involved in BR signal transduction but also involved in other signal pathways. To determine whether *BIG5* and *BIG3* is also involved in PIN1- and PIN2-mediated gravitropic response, PIN1-GFP and PIN2-GFP polarity were assayed in *big3-1 big5-1* and wild-type plants. As shown in Figure S10, PIN1-GFP and PIN2-GFP polar localizations were altered in *big3-1 big5-1* double mutants. In *big3-1 big5-1*, the PIN1-GFP displayed diffused location in vascular tissue, whereas PIN2-GFP showed abnormal location at the lateral side of epidermal cells. 

## 4. Materials and Methods

### 4.1. Contacts for Reagent and Resource Sharing

Further information and requests for resources and reagents should be directed to and will be fulfilled by the Lead Contact, Jie Le (lejie@ibcas.ac.cn).

### 4.2. Growth Conditions and Subject Details

#### 4.2.1. Growth Conditions

For analysis of seedling phenotypes, seeds were surface-sterilized in an aqueous solution of 30% (*w*/*v*) hydrogen peroxide and 85% (*v*/*v*) ethanol at a ratio of 1:4 (*v*/*v*). Then the seeds were sown on 0.8% agar-solidified half-strength Murashige and Skoog (MS) medium supplemented with 1% sucrose (pH 5.9). The plates were incubated at 4 °C for 36 h and then the seedlings were grown in a vertical orientation in growth champers at 21 °C under a 16-h light and 8-h dark cycle unless otherwise indicated.

#### 4.2.2. Genetic Materials

Transgenic *Arabidopsis thaliana* lines harboring following constructs have been described previously: *BRI1-GFP* [30,31], *BZR1-CFP* [17,41], *BZR1-1D-CFP* [41], *PIN1-GFP* [4,55], *PIN2-GFP* [15,56].

To generate *BIG5-MYC*, the full-length encoding sequence of *BIG5* was amplified by PCR amplified with the appropriate primers (see Table S1) from Arabidopsis cDNA. The purified PCR product was first cloned into entry vector pEasy-Blunt and then integrated to destination vector pSuper1300-221 with MYC tag. 

The engineered BFA-resistant *BIG5^M731L^–MYC* was constructed through primer-extension PCR with the appropriate primers (see Table S1). The purified PCR product was first cloned into entry vector pEasy-Blunt and then integrated to destination vector pSuper1300-221 with MYC tag.

To generate *pBIG1:GUS*, *pBIG2:GUS*, *pBIG3:GUS*, *pBIG4:GUS*, and *pBIG5:GUS*, the promoter rejoin of BIG family was amplified by PCR from Arabidopsis genomic DNA using primers listed in Table S1. The purified PCR product was first cloned into entry vector pEasy-Blunt and then integrated into destination vector 1300-221 with GUS tag or GFP tag to replace the 35S promoter.

To generate *pBIG5:BIG5-GFP* and *pBIG5:BIG5^M731L^–GFP*, the full-length encoding sequence *BIG5* and *BIG5^M731L^* was first cloned into entry vector pEasy-Blunt and then integrated to engineered vector 1300-221 with BIG5 promoter in N terminal and GFP tag in C terminal.

### 4.3. Methods

#### 4.3.1. RNA Isolation and Quantitative Real-Time PCR Analysis

Total RNA was extracted from 7-day-old seedlings using Plant Total RNA isolation kit (Magen-company, Guangzhou, China) according to the manufacturer’s protocol. RNA (5 μg) was treated with RNase-free DNase (Takara-company, Beijing, China), and 1 μg RNA was then used for cDNA synthesis using an iScript cDNA synthesis kit (Promega-company, Beijing China). ACTIN2 was used as the reference gene for all experiments. Quantitative real-time PCR analyses were performed with a Bio-rad CFX Connect instrument and relative expression values were calculated using the Delta-Delta-Ct (ddCt) Algorithm to calculate relative expression values of genes of interest. Real-time PCR primers are listed in Table S1.

#### 4.3.2. Chemical Treatments

BFA (Sigma-Aldrich, Saint Louis, MO, USA) were prepared as 50 mM stock in DMSO. eBL (Sigma-Aldrich, Saint Louis, MO, USA) were prepared as 10 mM stock in ethanol. Bikinin (MCE, Monmouth Junction, NJ, USA) was prepared as 50 mM stock in DMSO.

For BFA treatment, 5-day-old seedlings were immerged in half-strength liquid MS medium containing 50 µM BFA for 30 min. 

For short-term eBL treatment, 5-day-old seedlings were immerged in half-strength liquid MS medium containing 10 or 100 nM eBL for 120 min.

To examine the eBL effect on CPD and DWF4, 5-day-old seedlings were treated with 100 nM eBL for 2 hours. The relative expression levels of CPD and DWF4 were then analyzed by qRT-PCR.

For long-term eBL treatment, seedlings were grown on solid medium containing 10 or 100 nM eBL for 5 days. For all treatments with chemicals on solid medium, chemicals were added to half-strength MS (with or without Sucrose) solid medium at 55 °C before pouring the plates.

For Bikinin treatment, 5-day-old seedlings of BZR1-CFP transgenic plants in Col, *big3-1 big5-1,* and BZR1-1D-CFP in *big3-1 big5-1* grown on 0.5 MS solid medium were immerged in half-strength liquid MS medium containing 50 µM Bikinin for 4 h.

For FM4-64 staining, 5-day-old seedlings were incubated in ice-cold half-strength liquid MS medium containing 5 μg ml^–1^ of the membrane-selective dye FM4-64 (Invitrogen, Carlsbad, CA, USA) for 2 min as described before [57,58]. Then the samples were incubated in half-strength liquid MS medium at room temperature at indicated time before imaging. 

All the stock solutions were diluted in half-strength liquid MS medium for treatment at the indicated concentrations.

All the information of reagents and resource are provided in Table S2.

#### 4.3.3. Curvature and Gravity Responses Analyses

To examine the eBL effect on root gravitropic response, 5-day-old seedlings grown on half-strength MS medium supplemented with 10 nM eBL (without sucrose) were imaged and the root angle deviation from vertical was then analyzed using ImageJ [59].

To examine root gravity responses of BIG family gene mutants, 5-day-old seedlings grown on vertical plates were rotated 90° and photographs were taken at selected time points after reorientation. The angles of root curvature were calculated using the angles between root tip and gravity vector. 

To examine the effect of eBL on hypocotyl growth, the hypocotyl length of 3-day-old seedlings grown on vertical plates were measured using ImageJ [59].

#### 4.3.4. BZR1 Dephosphorylation Assay

BZR1-CFP transgenic plants in Col, *big3-1 big5-1*, and *BZR1-1D-CFP* in *big3-1 big5-1* background grown on half-strength MS medium for 5 days after germination (DAG) were incubated in half-strength liquid MS medium containing 10 or 100 nM EBL for 120 min. The plants were then harvested and ground to fine powder in liquid nitrogen. The tissues were extracted using a lysed buffer containing 50 mM PBS, 150 mM NaCl, 1% Triton X-100 (v/v), 1% glycerol (v/v), phosphatase inhibitor cocktail (Yeasen, Shanghai, China), and protease inhibitor cocktails (Roche, Basel, Switzerland) at 4 °C. The total protein was added 5× SDS loading buffer and boiled for 15 min. The cell lysate was cooled to 4 °C and centrifuged at 10,000 rpm for 15 min. Then 0.5 mL supernatant of total protein extract was added with 20 μL anti-GFP sepharose (MBL). After incubation at 4 °C for 1 h, the agarose was washed twice with lysed buffer. Samples were boiled in SDS loading buffer for 15 min and then separated by 12% SDS-PAGE gels, transferred to a PVDF membrane (Millipore, Massachusetts, USA) using a semi-dry blotting system (Bio-Rad, California, USA), and then incubated with an anti-GFP monoclonal antibodies followed by HRP-conjugated anti-mouse antibodies. The signals were detected using Super Signal West Dura chemiluminescence reagent (Pierce, Rockford, MI, USA) Kit.

#### 4.3.5. Confocal Laser-Scanning Microscopy and Quantitative Analyses of Fluorescence Intensity and Co-localization

For confocal imaging, samples were placed in water under a coverslip. Imaging was performed using a 40× water lens, on an OLYMPUS FV1000-MPE confocal microscope. An Ar+/Kr+ laser was used to excite GFP at 488 nm and RFP at 568 nm simultaneously. For imaging GFP, argon ion (488 nm) excitation laser was used, collected at 495–530 nm. For mCherry 561 nm excitation laser was used, collected at 600–620 nm for mCherry. A sequential mode of imaging was used to avoid nonspecific excitation.

#### 4.3.6. Quantification and Statistical Analysis of BFA Compartments

For quantification of BRI1-GFP aggregation in BFA-compartment, more than 130 epidermal cells from at least 5 root tips were analyzed. Number of BFA-compartment per cell and the percentage of cells carrying BFA-compartments were counted.

### 4.4. Accession Numbers

Sequence data from this article can be found in the GenBank/EMBL library under the following accession numbers: *ARF1* (At1g23490), *BIG1* (At4g38200), *BIG2* (At3g60860), *BIG3* (At1g01960), *BIG4* (At4g35380), *BIG5* (AT3G43300), *GNOM* (At1g13980), *GNL1* (At5g39500), *PIN1* (At1g73590), *PIN2* (AT5G57090), *BRI1* (AT4G39400), *BZR1* (AT1G75080), *VHA-a1* (At2g28520).

## 5. Conclusions

Taken together, the loss-of-function of *BIG5* reduced sensitivity to BRs. Our findings provided important and novel insights into understanding the complicated process of BRI1 recycling in BR signaling. It will be interesting to test how multiple ARF-GEFs coordinately regulate the endomembrane recycling-dependent BR signaling and auxin transport.

## Supplementary Materials

Supplementary materials can be found at https://www.mdpi.com/1422-0067/20/9/2339/s1. Figure S1 Physical maps of BIG-subfamily *ARF-GEF* genes and the relative transcript levels in mutants. Figure S2. Functional redundancy between BIG5 and BIG3 in regulating plant growth. Figure S3 *big3-1 big5-1* show a reduced sensitivity to eBL. Figure S4 BIG5 partially colocalizes with TGN marker VHA-a1-GFP and key residue within SEC7 domain determines the BFA sensitivity of ARF-GEFs. Figure S5 Expression patterns of BIG-subfamily ARF-GEF genes in young seedlings. Figure S6 *big5-1* and *big3-1 big5-1* mutants display a hypersensitive response to gravity stimulation. Figure S7 the negative gravitropic growth is suppressed by eBL but *big5* exhibits a reduced sensitivity. Figure S8 *big5-1* and *big3-1 big5-1* display a delayed internalization of FM4-64 dyes. Figure S9 Genetic relationship analysis of *BIG5* and *BRI1*. Figure S10 Mutations in *big3-1 big5-1* induce ectopic PIN1 and PIN2 subcellular localization. Table S1. Primers used in this study. Table S2. Detail of the key reagent and resource information.

## Abbreviations

ARF	ADP-ribosylation factor
ARF-GEF	ARF-guanine nucleotide exchange factor
BFA	Brefeldin A
BRs	Brassinosteroids
eBL	Epibrassinolide
GFP	Green fluorescent protein
GUS	β-Glucuronidase
TGN	Trans-golgi network
WT	Wild-type
