# Supplementary material for: Involvement of BIG5 and BIG3 in BRI1 Trafficking Reveals Diverse Functions of BIG-subfamily ARF-GEFs in Plant Growth and Gravitropism
Source: Int J Mol Sci. 2019 May 11;20(9):2339. doi: 10.3390/ijms20092339 (PMC6539719; doi:10.3390/ijms20092339)
Supplement: Supplementary file 1 [file ijms-20-02339-s001.zip › ijms-500144 suppl/ijms-500144 suppl figures.pdf]

**Fig.** S1

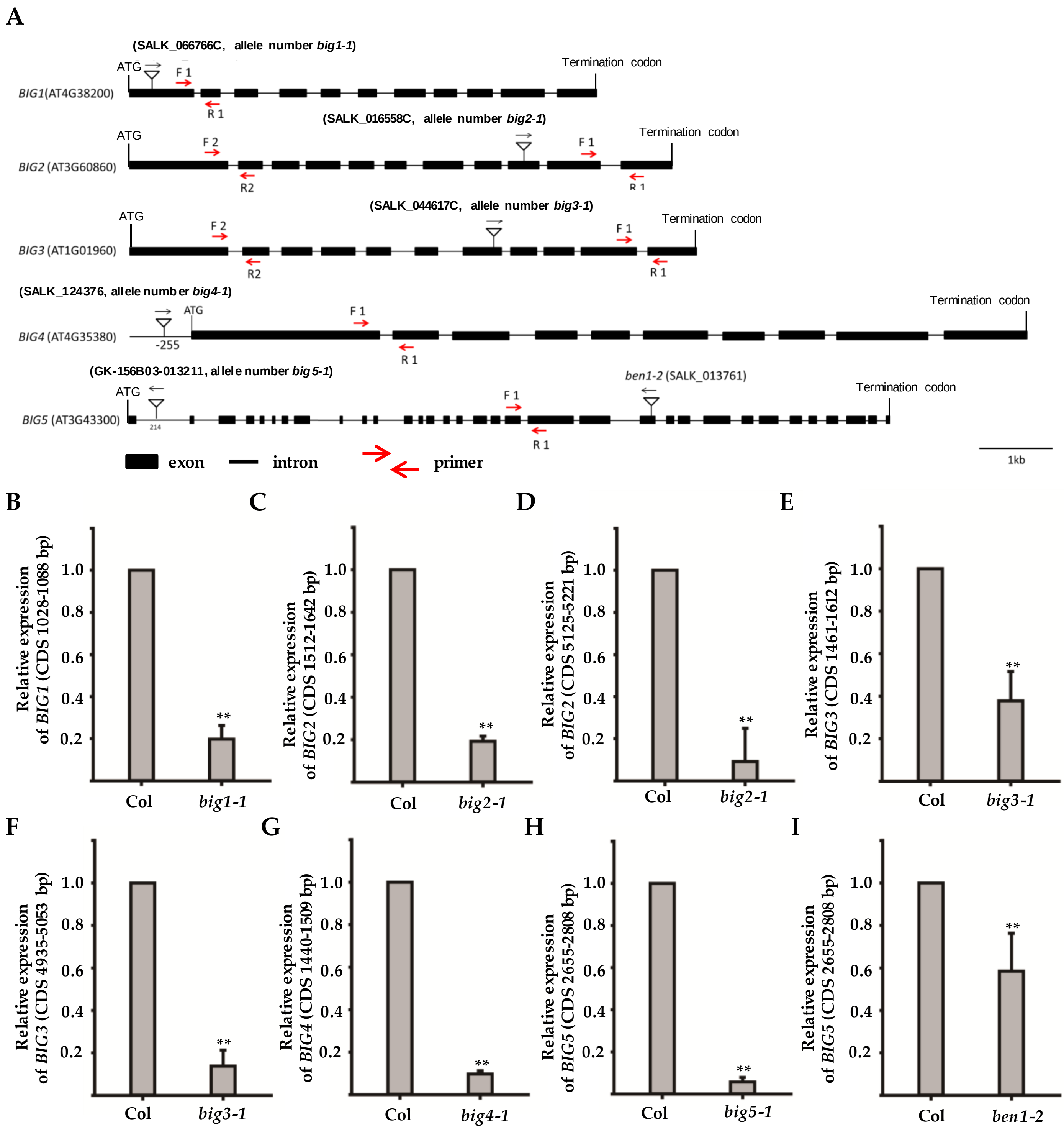


**Figure S1** Physical maps of *BIG*-subfamily *ARF-GEF* genes and the relative transcript levels in mutants. (A) Physical maps. (B-I) Transcription levels. Red arrows indicate the position of primers used for RT-qPCR assays. Error bars represent standard deviations, significant difference after Student's *t*-test, $^{**}P<0.01$. Two pairs primers used for *BIG3* transcription assay. *big1-1* contains a T-DNA insertion between 110 bp to 400 bp, primers No.1 in table S1 used to test for *big1* homozygosity, No.2 for T-DNA insertion identify. *big2-1* contains a T-DNA insertion between 4587 bp to 5044 bp, primers No.3 in table S1 used to test for *big2* homozygosity, No.4 for T-DNA insertion identify. *big3-1* contains a T-DNA insertion between 4436 bp to 4440 bp, primers No.5 in table S1 used to test for *big3* homozygosity, No.6 for T-DNA insertion identify. *big4-1* contains a T-DNA insertion between -255 bp to -128 bp, primers No.7 in table S1 used to test for *big4* homozygosity, No.8 for T-DNA insertion identify. *big5-1* contains a T-DNA insertion between 191 bp to 314 bp, primers No.9 in table S1 used to test for *big5* homozygosity, No.10 for T-DNA insertion identify.

**Fig. S2**

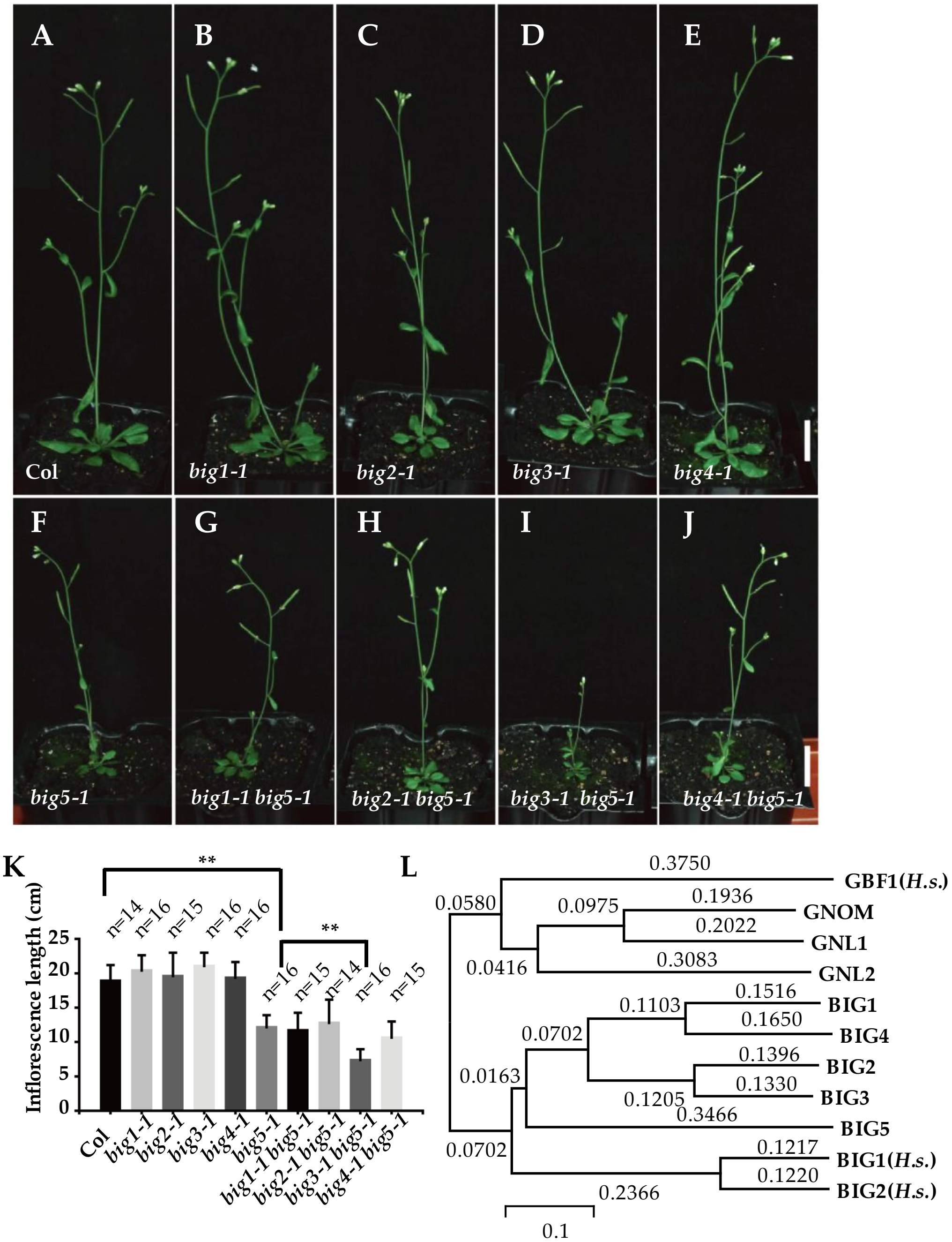


**Figure S2. Functional redundancy between BIG5 and BIG3 in regulating plant growth.**
(A-F) Six-week-old plants of wild type (A) and *BIG*-subfamily single mutants (B-F). Among single mutants , *big5-1* showed a reduced rosette size and inflorescence length. (G-J) Six-week-old plants of *big1-1 big5-1*, *big2-1 big5-1*, *big3-1 big5-1*, and *big4-1 big5-1* double mutants. A severely retarded growth only observed in *big3-1 big5-1* double mutants. Bars= 3 cm. (K) Quantitative analysis of inflorescence length. Error bars represent standard deviations, significant difference after Student's *t*-test, $^{**}P<0.01$. (L) ARF-GEFs full length CDS of *Arabidopsis thalina* and *Homo sapiens* were aligned using Clustal W and the phylogenetic tree drawn with MEGA. Arabidopsis GBF1-subfamily ARF-GEF are conserved corresponding to human GBF1-subfamily ARF-GEF, while Arabidopsis BIG-subfamily conserved to human BIG1/2.

**Fig. S3**

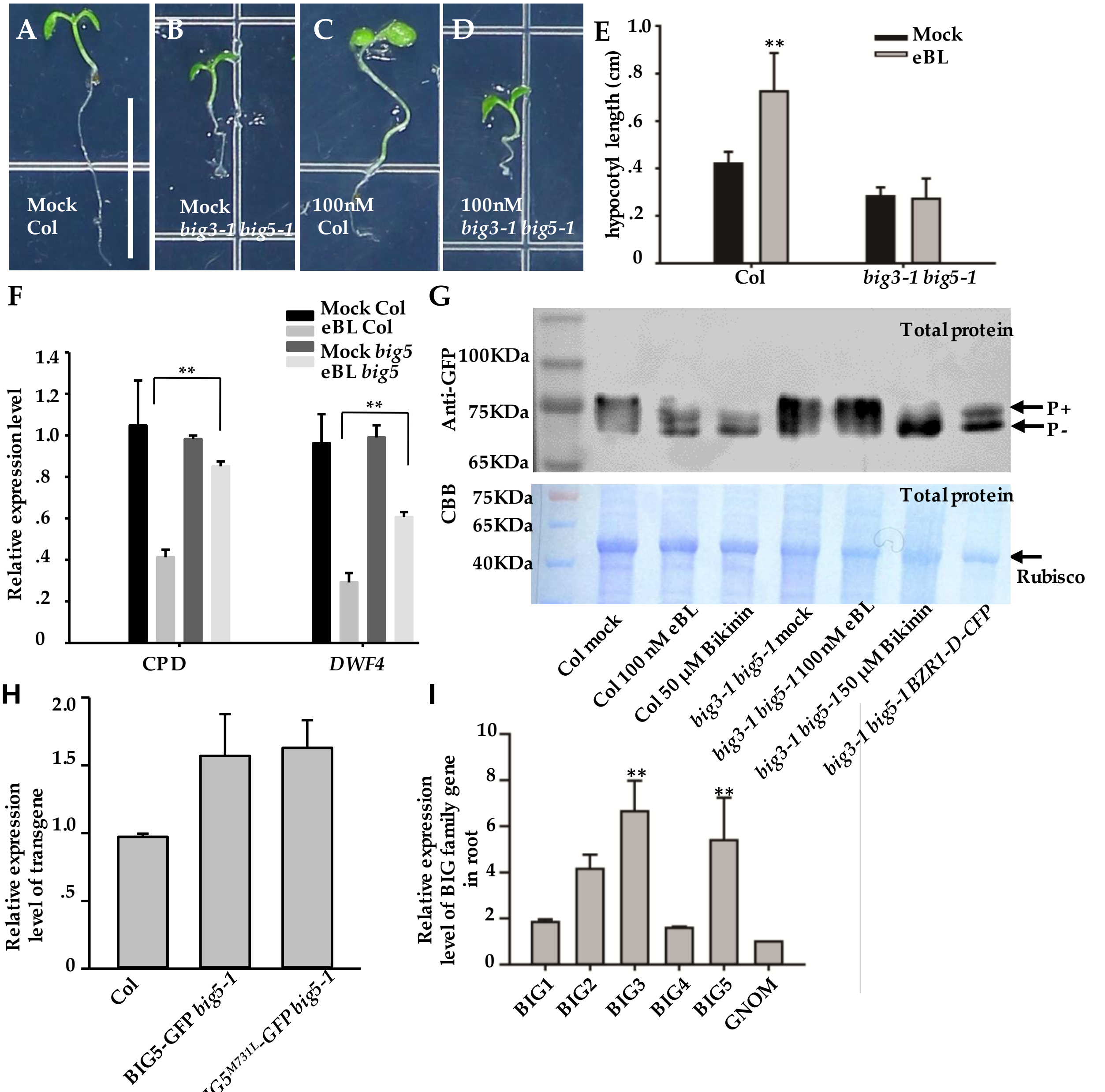


**Figure S3** ***big3-1 big5-1*** **show reduced sensitivity to eBL**

(A- E) Seedlings at six day after germination grown on 1/2MS (plate vertically placed) without or with 100 nM eBL. *big3-1 big5-1* hypocotyls display a reduced sensitivity to eBL. (F) The expression levels of a BR marker gene *CPD* and *DWF4* reduced response to 100 nM eBL in *big3-1 big5-1* mutant compared Col. (G) Comparing with Col, the BZR1-CFP dephosphorylation is reduced in *big3-1 big5-1* in response to eBL. By contrast, the BZR1-CFP dephosphorylation in both Col and *big3-1 big5-1* is promoted by Bikinin. Rubisco in the bottom CBB gel used as the loading control. (H) *BIG5-BIG5-GFP* and *BIG5-BIG5*$^{M731L}$*-GFP* display a comparable expression level of transgenes. (I) The expression levels of BIG family genes in roots.

Scale bars=1 cm in panel A-D; Error bars represent standard deviations, significant difference after Student's *t*-test, $^{**}P$<0.01.

**Fig. S4**

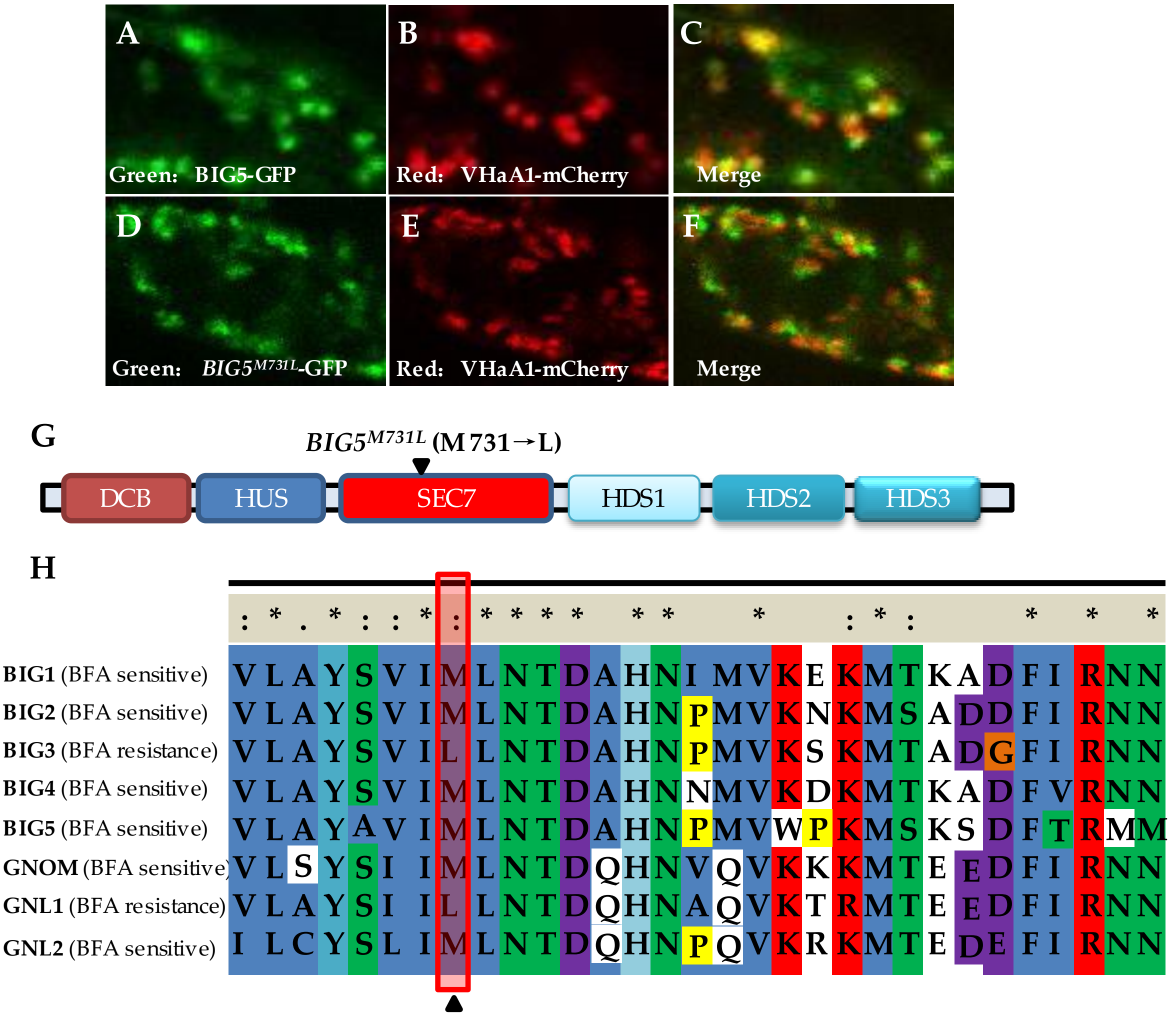


**Figure S4 BIG5 partially colocalizes with TGN marker VHA-a1-GFP and key residue within SEC7 domain determines the BFA sensitivity of ARF-GEFs.**

(A-F) BIG5-GFP and BIG5$^{M731L}$-GFP partially colocalize with TGN marker VHA-a1-GFP. (G) Mutation of BFA-resistant *BIG5$^{M731L}$* is located within the SEC7 domain. (H) Alignment of the motifs of SEC7 domain from ARF-GEFs using ClustalW. The red frame indicates the key amino acid for the BFA sensitivity. Substitution of M731 turns the BFA-sensitive BIG5 into BFA-insensitive.

**Fig S5**

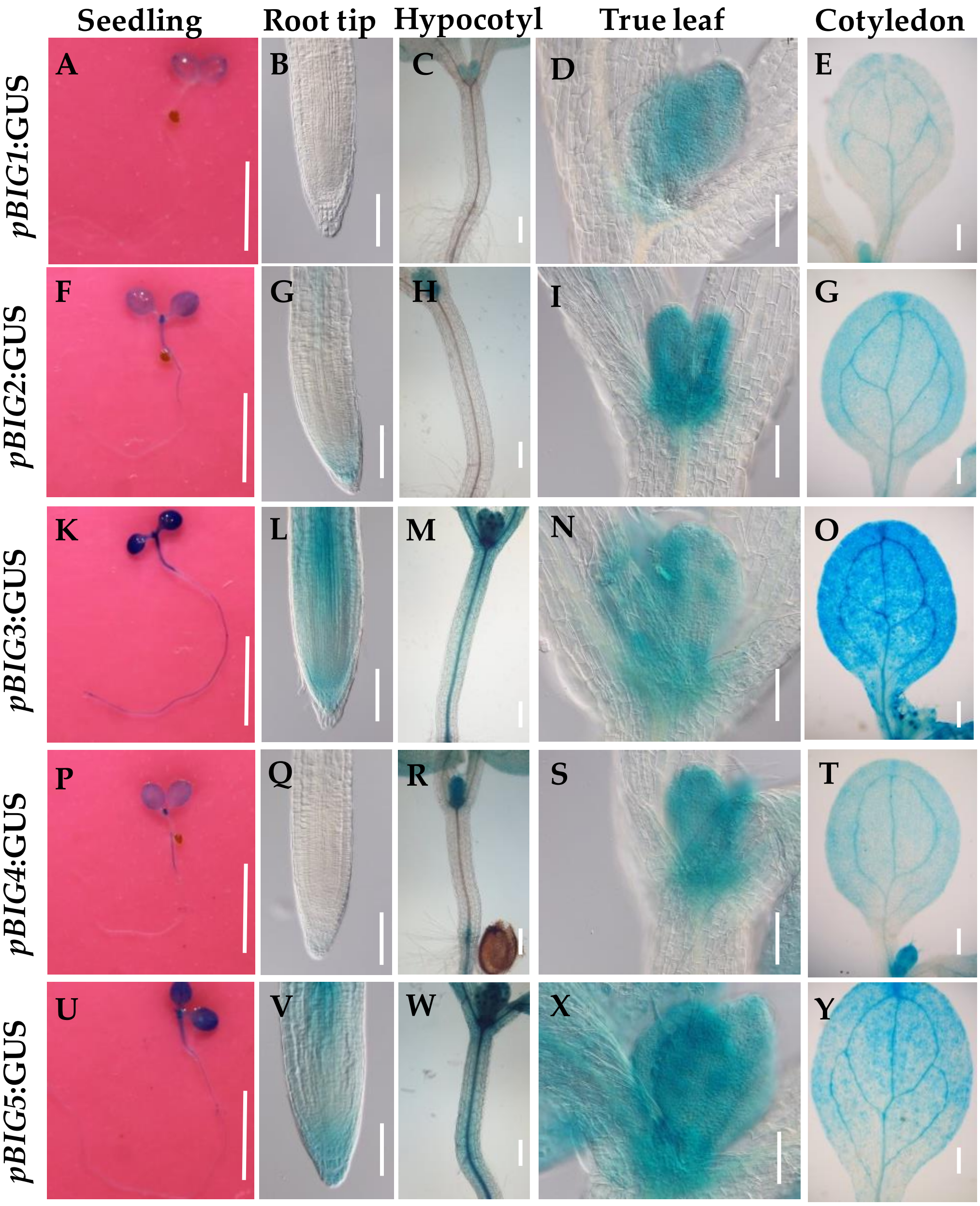


**Figure S5 Expression patterns of *BIG*-subfamily *ARF-GEF* genes in young seedlings.**

(A-E) *BIG1* gene mainly expresses in cotyledons and young true leaves. (F-J) *BIG2* gene shows weak expression in roots, cotyledons, and young true leaves. (K-O) *BIG3* gene has strong expression in all developing organs and hypocotyl. (P-T) *BIG4* gene mainly expresses in cotyledons and young true leaves. (U-Y) *BIG5* gene has strong expresses in all developing organs especially in root tips and hypocotyls. Seedling at five days after germination were used for GUS staining. Bars=0.5 cm in panel A, F, K, P, U; 100 μm in the rest panels.

**Fig S6**

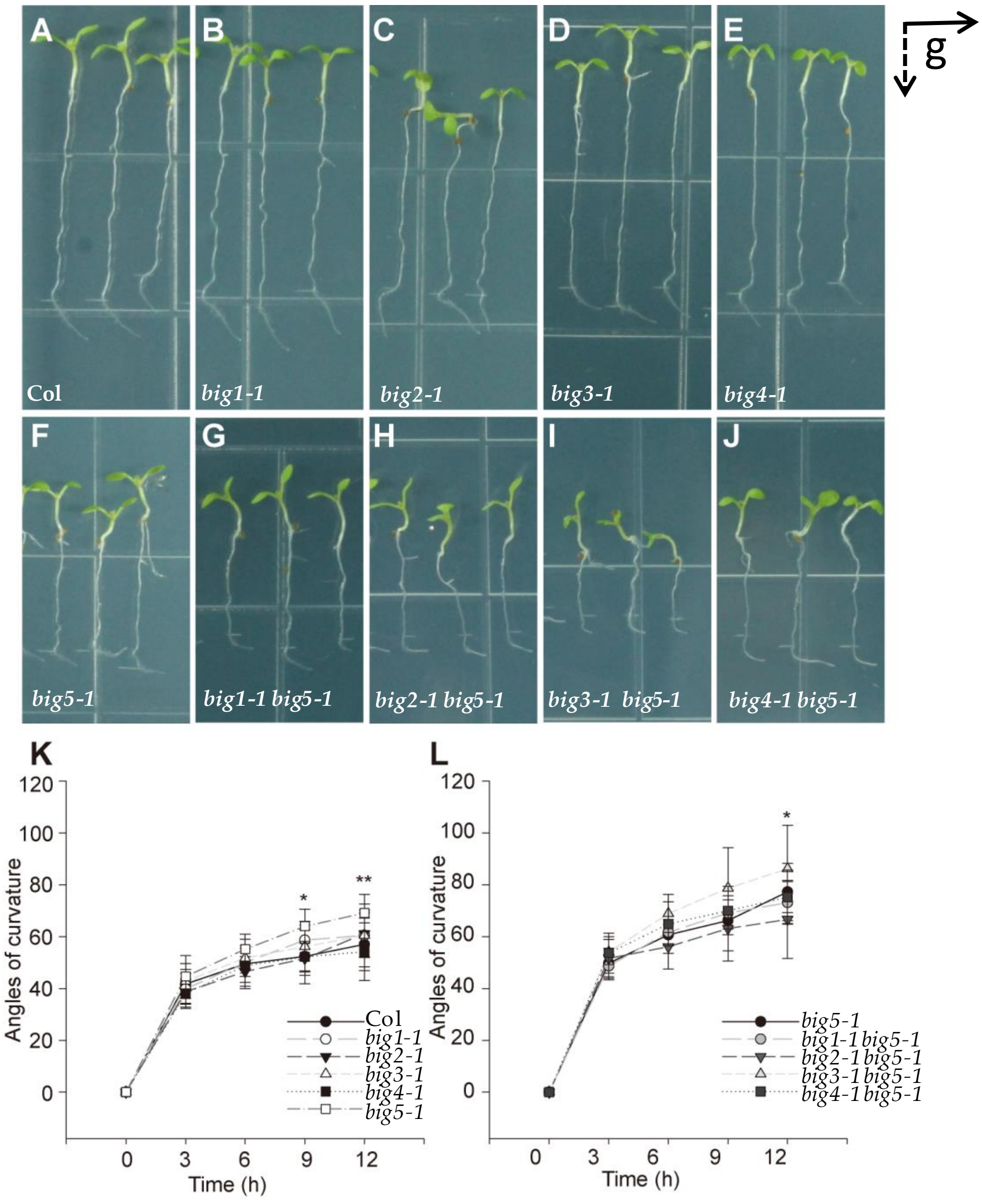


**Figure S6** ***big5-1*** **and t*big3-1 big5-1* mutants display a hypsensitive response to gravitropic stimulation.** (A-F) The root gravitropic response of BIG-subfamily single mutants (B-F) compared with wild-type (A). The gravitropic response was significantly accelerated in *big5-1* single mutant (F). Quantitative analysis is shown in (K). (G-J) The root gravitropic response of BIG-subfamily double mutants. *big3-1 big5-1* (I) enhanced *big5-1* (F) phenotype in root length and gravitropic response. Quantitative analysis is shown in (L). The angles of the root growth direction against gravity vector when the agar plate had been reoriented 90° (5 d after germination). The scale bar represents 1.5 cm, Error bars represent standard deviations, significant difference after Student's *t*-test, asterisk means *$P$<0.05, **$P$<0.01.

## Fig S7

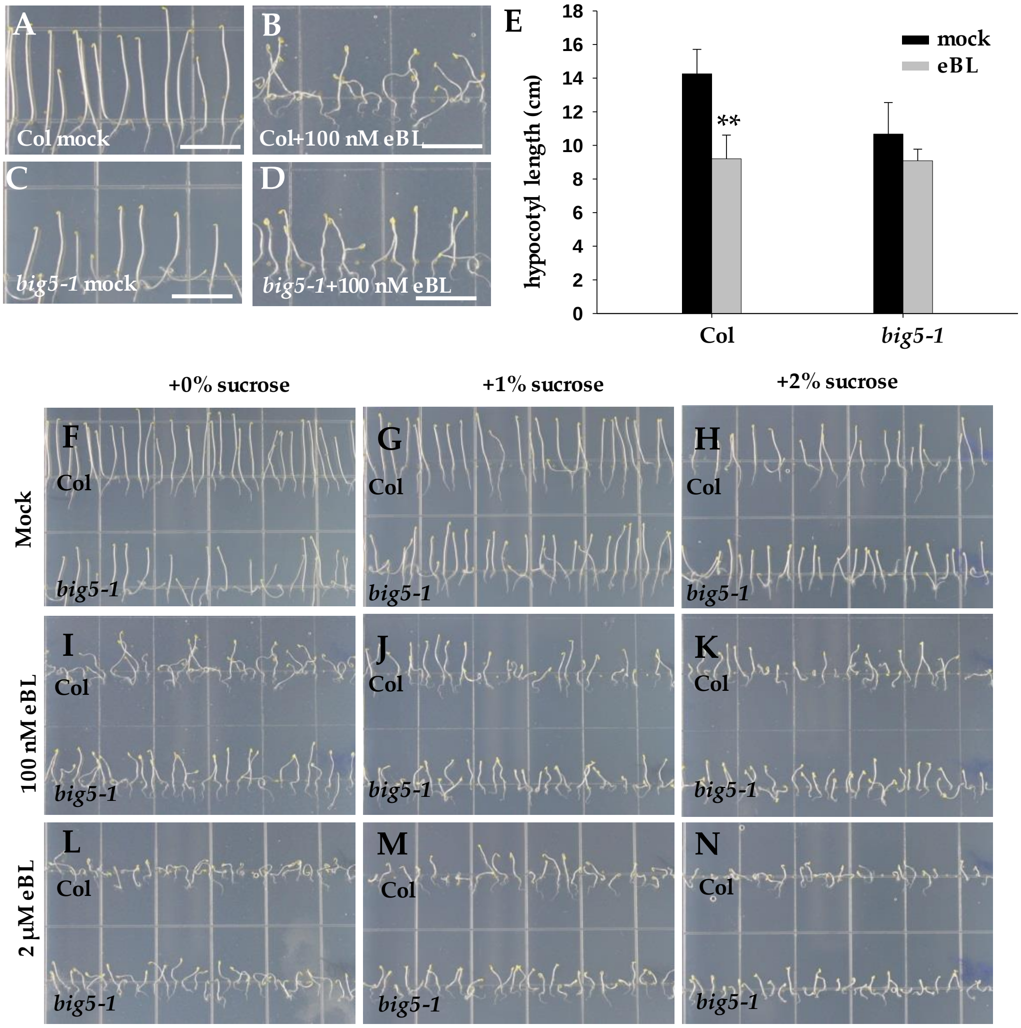


**Figure S7 The negative gravitropic growth is suppressed by eBL but *big5* exhibits a reduced sensitivity.**

(A-E) Etiolated hypocotyls at three day after germination grown on 1/2MS (plates were vertically placed) without or with eBL. Elongation of hypocotyls are inhibited by eBL in Col but not in *big5-1*. Scale bar=1 cm. Error bars represent standard deviations, significant difference after Student's *t*-test, $^{**}P<0.01$. (F-H) Both Col ane big5-1 mutant show negative gravitropic growth (vertical growth). (I-K) Etiolated hypocotyls grown on 100 nM eBL-supplemented medium show twisted and non-vertical growth. Supplement of sucrose could reduced the eBL effects. (L-N) The vertical growth and elongation of hypocotyls are severely suppressed by 2 μM eBL. Compared with Col., *big5-1* showed a reduced sensitivity to eBL

**Fig. S8**

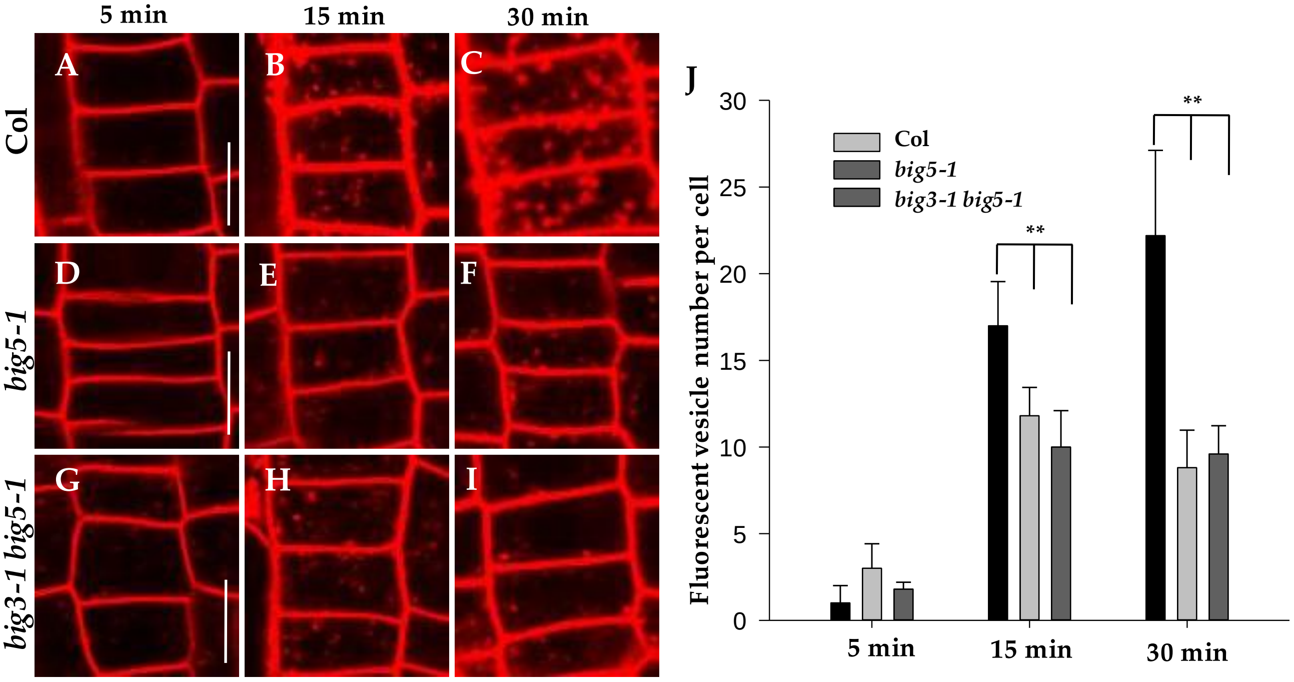


**Figure S8 *big5-1* and *big3-1 big5-1* display a delayed internalization of FM4-64 dyes.**

(A-C) The punctate FM4-64 fluorescent vesicles obviously appeared inside the cells at 15 min and increased at 30 min after dye incubation. (D-I) The number of visible FM4-64 fluorescent vesicles inside cells of *big5-1* and *big3-1 big5-1* mutants are much less that that in Col. (J) Quantitative analysis the number of visible FM4-64 fluorescent vesicles per cell.

Bars=10 μm. Error bars represent standard deviations, significant difference after Student's *t*-test, $^{**}P<0.01$.

**Fig. S9**

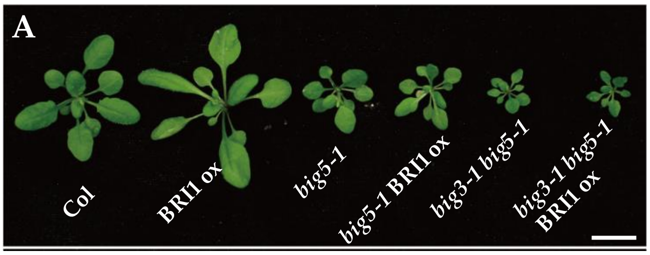


**Figure S9 The given function of BRI1 OX suppressed in *big5-1* and *big3-1big-5*.**

(A) *BRI1* overexpression (*BRI1:BRI1-GFP*) causes an increase of plant size, but this effect is suppressed either in *big5-1* or in *big3-1 big5-1* mutants. Scale bar=1 cm.

**Fig. S10**

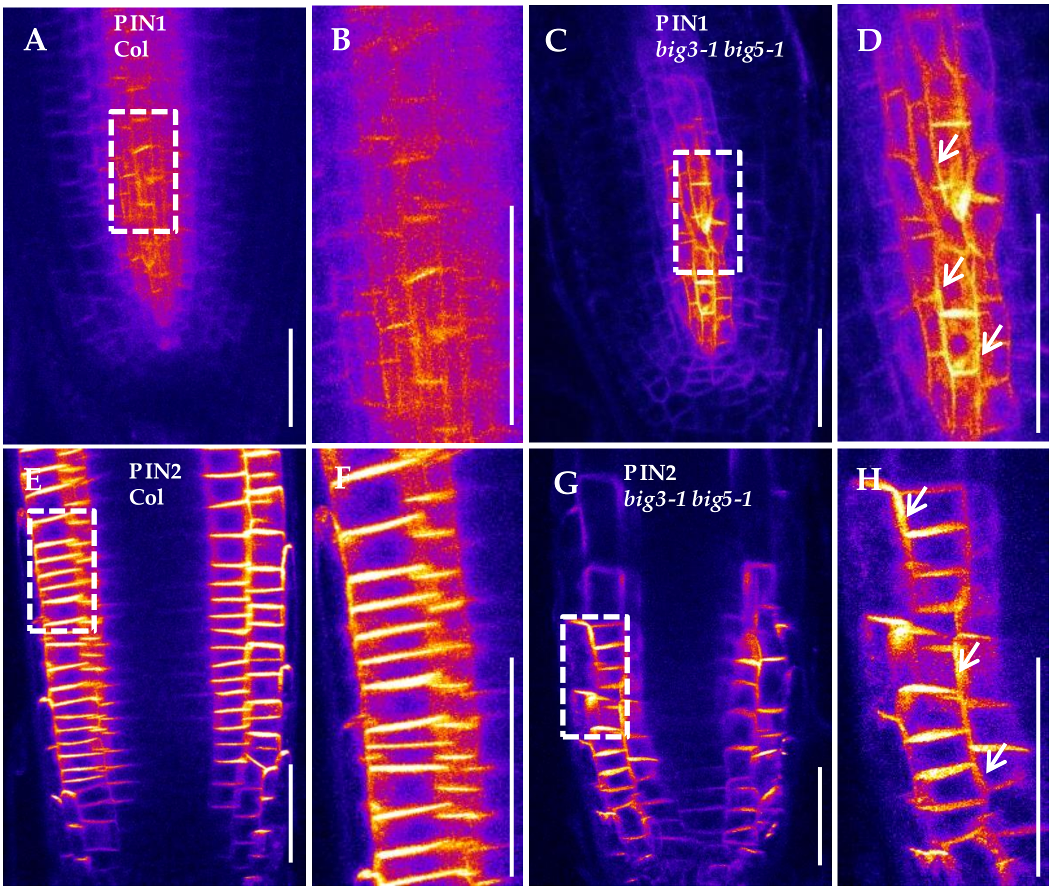


**Figure S10 Mutation in *big3-1 big5-1* induces ectopic PIN1 and PIN2 subcellular localization.**

(A) In Col, PIN1-GFP shown a polar localization at bottom of cells. (B) Enlarged region within the white frame in panel A. (C) In *big3-1 big5-1* double mutant, PIN1-GFP showed an ectopic localization. (D) Enlarged region within the frame in panel C. (E) In Col, PIN2-GFP location in top of epidermal cells. (F) Enlarged region within the frame in panel E. (G) In *big3-1 big5-1* mutant, PIN2-GFP showed ectopic localization at the lateral side of epidermal cells. (H) Enlarged region within the white frame in panel G.

Scale bars=40 μm. White arrows point to the ectopic localization of PIN1-GFP or PIN2-GFP.
