# Supplementary material for: Involvement of BIG5 and BIG3 in BRI1 Trafficking Reveals Diverse Functions of BIG-subfamily ARF-GEFs in Plant Growth and Gravitropism
Source: Int J Mol Sci. 2019 May 11;20(9):2339. doi: 10.3390/ijms20092339 (PMC6539719; doi:10.3390/ijms20092339)
Supplement: Supplementary file 1 [file ijms-20-02339-s001.zip › ijms-500144 suppl/ijms-500144 suppl tables.pdf]

Table S1. Primers used in this study

No.	Name	Forward (5'-sequence-3')	Reverse (5'-sequence-3')	Note
1	*big1-1*	GGATTAGGATGCGAGGAGATC	CGGTGATAAAATTGTTGGACG	wild-type identify
2	*big1-1*	GGATTAGGATGCGAGGAGATC	TGGTTCACGTAGTGGGCCATCG	T-DNA insertion identify
3	*big2-1*	TTTCCCACTTTTTCCACTGTG	TTGAGGGGTTCATATGACAGC	wild-type identify
4	*big2-1*	TTTCCCACTTTTTCCACTGTG	TGGTTCACGTAGTGGGCCATCG	T-DNA insertion identify
5	*big3-1*	AAACTCTCCACTGGCTAAGCC	GCAAGTTTTCTTGCGCAATAC	wild-type identify
6	*big3-1*	AAACTCTCCACTGGCTAAGCC	TGGTTCACGTAGTGGGCCATCG	T-DNA insertion identify
7	*big4-1*	AAACCCACAATAATCTATGGCC	AATCGGTTCTTCGGCTATAGC	wild-type identify
8	*big4-1*	AAACCCACAATAATCTATGGCC	TGGTTCACGTAGTGGGCCATCG	T-DNA insertion identify
9	*big5-1*	CCGTCGTATCTCCGACGATCTT	CCCCGAAGATGACAAGTTCAA	wild-type identify
10	*big5-1*	CCGTCGTATCTCCGACGATCTT	GGGCTACACTGAATTGGTAGCTC	T-DNA insertion identify
11	*BIG1*	TCATTGATAACGGCGGCCCA	ACAGCGACAAGCAGAGCAACT	For BIG1 expression level test
12	*BIG2*	CTAAGTGCCGAGCCGCTGTT	CCGTGCAATGCATCTACGAGGA	For BIG2 expression level test, N terminal
13	*BIG2*	TCGGCAGATCCGCAGTCGAT	AGCACTGTTCTTCAACAACGACA	For BIG2 expression level test, C terminal
14	*BIG3*	TGCTGTCGTCACAGATGCCA	ATGCCGTGCATAGCGTCGAA	For BIG3 expression level test, N terminal
15	*BIG3*	TGAGAATGCCGGTGCTGTCT	GCCTTCAGCCCAGCACGAAA	For BIG3 expression level test, C terminal
16	*BIG4*	ACATACTTGAAAGGATTGTCAACGG	ATCTTGCGCTGGAGACAACG	For BIG4 expression level test
17	*BIG5*	CTGCATGGAGGGATTTAAAGCTGGA	TCTGAGTCACACAACCCCAGT	For BIG5 expression level test

18	*BIG3*	ATGGCTTCTACGGAAGTCGATTT	GCAGCAAGAGCGGAGGAGAACA	For amplification BIG3 coding sequence
19	*BIG5*	ATGGCGGCTGGTGGATTTTTGA	CTGTTGCAAAAGTGGCTTC	For amplification BIG5 coding sequence
20	*BIG5^{M731L}*	AGCCTATGCAGTTATCTTATTAAATACA	GATTATGCGCATCTGTATTTAATAAGAT	For introducing an ATG (M) to CTG (L) mutate in BIG5 SEC7 domain
21	*pBIG1*	TTACCTTCTTGTTGATTTTGGCA	CTAACGCATATTCGACTTCTCAC	For amplification BIG1 promoter rejoin
22	*pBIG2*	CACACATCTCGTTATGCATACCC	TCCCAATTTTCACGGCGAGCTCA	For amplification BIG2 promoter rejoin
23	*pBIG3*	GGCCGTATGATTTCACATGAACC	TCCAGATTGGTAGCCGTGACTTC	For amplification BIG3 promoter rejoin
24	*pBIG4*	CGACTCCCATTTGAAATGATCCG	CGCCGGAAAATCAAAAGTTTTGA	For amplification BIG4 promoter rejoin
25	*pBIG5*	GAAAAGCTTTGAACTGTCCCAC	CTTTAATCCTTCCTATTTCGACT	For amplification BIG5 promoter rejoin

Table S2. Detail of the key reagent and source information

REAGENT or RESOURCE	SOURCE	IDENTIFIER
**Antibodies**		
anti-CFP monoclonal antibody	pierce	CAT#PA526939
HRP-conjugated anti-mouse antibody	Abcam	CAT#ab19195
**Chemicals, Peptides, and Recombinant Proteins**		
BFA	Sigma-Aldrich	CAT#87022601
eBL	Sigma-Aldrich	CAT#E1641-2MG
RNase-free DNase	Takara	CAT#2270A
protease inhibitor cocktails	Roche	CAT#4693116001
FM4-64	Invitrogen	CAT#F34653
Bikinin	MCE	CAT#HY-12524
InStab^{TM} Phosphatase Inhibitor Cocktail	Yeasen	CAT#20109ES05
**Critical Commercial Assays**		
Plant Total RNA isolation kit	Magen	CAT#MD5215-01
iScript cDNA synthesis kit	Promega	CAT#A6001
Super Signal West Dura chemiluminescence reagent Kit	Pierce	CAT#34075
**Experimental Models: Organisms/Strains**		
BRI1-GFP	Friedrichsen et al., 2000; Wang et al., 2001	N/A
BZR1-CFP	Wang et al., 2002	N/A
BZR1-D-CFP	Wang et al., 2002	N/A
PIN1-GFP	Friml et al., 2002; Benkova´ et al., 2003	N/A
PIN2-GFP	Xu and Scheres, 2005; Blilou et al., 2005	N/A
ST-RFP	Naramoto, 2014	N/A
VHA-a1-RFP	Naramoto, 2014	N/A
BIG5-GFP	This paper	N/A
BIG5^{M731L}-GFP	This paper	N/A
pBIG1:GUS	This paper	N/A
pBIG2:GUS	This paper	N/A
pBIG3:GUS	This paper	N/A
pBIG4:GUS	This paper	N/A
pBIG5:GUS	This paper	N/A
**Recombinant DNA**		
pEasy-Blunt vector<br>pSuper1300-221 vector<br>pCAMBIA1300-221 vector	Transgene	N/A
**Software**		
ImageJ	http://rsbweb.nih.gov/ij/	http://rsbweb.nih.gov/ij/
